# Environments for Active Lifestyles: Sustainable Environments May Enhance Human Health

**DOI:** 10.4137/EHI.S1037

**Published:** 2008-10-30

**Authors:** Takemi Sugiyama

**Affiliations:** Cancer Prevention Research Centre, University of Queensland, Brisbane, Australia

## Introduction

A growing number of studies focus on the role of environments in promoting active lifestyles.^1^ Being physically active in daily life, which is known to have significant health benefits,^2^ is easier to do in some environments than it is in others. Despite mounting evidence on the relationships between environmental characteristics, activity patterns, and health,^3^ most decisions on environmental planning and design appear to be made without considering their implications for residents’ activity and their health.

In order to promote physically-active lifestyles through environmental design, it is desirable that environmental decision-making processes explicitly incorporate people’s activity patterns as a key criterion. However, those who are involved in environmental planning and design are not necessarily aware that their decisions could ultimately affect people’s health by influencing their behaviors. Although the importance of collaboration between planning, transportation and public health has been advocated for many years,^1^ there remains significant work to be done to realize such cooperation.

This commentary discusses the possibility of making use of existing planning and design initiatives to promote active lifestyles. More specifically, I argue that environmental planning principles aiming for sustainability in urban or suburban areas, such as compact city and smart growth, could not only make environments conducive to physical activity, but also help reduce time in sedentary behavior—newly identified and significant health risk. Human health and environmental sustainability are both top-priority issues in today’s society. The planning and design of environments that can improve human and environmental health simultaneously are more likely to receive strong support from a wide range of stakeholders, thus have a better chance of being implemented.

## Health and Active Lifestyles

Active lifestyles involve two distinct types of behaviors: physical activity and sedentary behavior (sitting). Ample evidence demonstrates significant health benefits of physical activity. Regular participation in moderate-intensity physical activity reduces the risks of cardiovascular disease, type 2 diabetes, breast and colon cancers, several other chronic illnesses, and mental illness.^2^,^4^ Despite the known benefits of physical activity and public health efforts to promote activity, the proportion of people who meet the current guideline of physical activity is low; in Australia, 70% of those over 15 years of age do not reach the recommended level of physical activity.^5^ Given the increasing prevalence of the ‘diseases of inactivity’, increasing physical activity has become a public health priority.^6^

Our lifestyles are becoming increasingly dominated by sedentary behaviors, due in no small part to the advent of technologies that allow or force us to sit, including automobiles, television sets, computers, and the Internet.^7^ Recent research has shown that leisure-time sedentary behavior, typically TV viewing time, is associated with metabolic biomarkers (elevated blood glucose levels, triglycerides, and waist circumference) that are significantly related to the risk of diabetes and cardiovascular disease.^8^ Driving for commuting is another common sedentary behavior that is shown to have a negative impact on health by increasing the likelihood of overweight and obesity.^9^ It has to be noted here that physical activity and sedentary behavior are independently associated with health outcomes; that is, prolonged sitting time contributes to poor health, regardless of leisure-time physical activity levels.^10^

It is important to stress that sedentary behavior is not necessarily the same as a lack of physical activity: an individual can be sufficiently physically active (meeting public health guidelines on the recommended level of physical activity, 30 minutes/day of moderate-intensity physical activity for 5 days/week), yet still spend a considerable amount of time in sitting (at work, for transport, and during leisure time). Conversely, those who do not participate in physical activity could nevertheless engage in high volumes of light-intensity activities, thus spending less time in sedentary behaviors. Thus, increasing physical activity and reducing sedentary behavior are each distinct and important strategies for achieving active lifestyles and associated health benefits.

## Active Lifestyles and the Environment

Neighborhood environments play an important role in active lifestyles. Physical activity does not have to be structured, planned exercise: it can be incidental activity such as brisk walking for transport or for recreation.^4^ Although structured vigorous ‘exercising’ does have additional health benefits, moderate-intensity activity happening daily in a neighborhood environment is considered to be important because such activity can be easily embedded into one’s everyday life patterns, and thus more likely to be maintained in the longer term. There is another reason for addressing environmental attributes to promote active lifestyles. Research has shown that individual-level interventions to increase physical activity, which employ educational, behavioral, and cognitive strategies, tend to be effective in a short term, but are less successful in bringing about longer-term maintenance of activity patterns.^11^ This has led researchers to pay attention to contextual factors that should support active lifestyles.

A growing body of literature has examined environmental correlates of physical activity. It can be argued from research so far that important environmental characteristics that facilitate physical activity are the availability of various *destinations* nearby (both utilitarian and recreational) and the quality of *routes* to such destinations. For example, the number of commercial destinations in a neighborhood has been found to be associated with walking for transport.^12^ A greater mixture of land uses (residential, commercial, recreational) and a good facility for walkers and cyclists were shown to contribute to residents’ physical activity for transport and for recreation.^13^ Neighborhood aesthetics was also found to be relevant to physical activity levels.^14^

In contrast to the plethora of research on the environmental correlates of physical activity, little research has examined environmental attributes associated with sedentary behavior. However, in the case of driving for commuting or for shopping, it would seem logical that a lack of destinations within a walking distance and lack of access to public transport would be major factors contributing to the choice of using an automobile, as opposed to active modes of transport such as walking or bicycling.

Thus, environments conducive to active lifestyles ideally have the following characteristics:

- high residential density and mixed land use, which make various destinations such as shops and services close enough for walking or bicycling
- accessible public transportation systems that help reduce private vehicle use, and encourage activity for transport
- availability of recreational spaces, such as parks, community gardens, play grounds, and river banks, which entice recreational physical activity
- good walking and cycling infrastructure with attractive surroundings

Recreational green spaces in neighborhoods (such as parks) merit consideration. In addition to providing opportunities for physical activity, such spaces enable people to have contact with nature. Research has shown that contact with green, natural elements has “restorative” or “therapeutic” effects. For instance, it has been found that the amount of time people spend in green areas is associated with a reduced risk of stress-related illnesses.^15^ Walking in a natural setting, compared to walking in a built-up urban setting, is also found to have a positive effect on mental health.^16^ Thus, contact with nature, particularly in combination with being physically active, is another environment-related factor that can be a significant contributor to better health.

## Sustainable Environments for Active Lifestyles?

It is remarkable that these environmental qualities relevant to active lifestyles (and contact with nature) are almost identical to characteristics considered essential to achieve environmental sustainability. Compact urban form (achieved by high residential density and mixed land use) and public transport would reduce energy consumption and greenhouse gas emission mainly by minimizing the trips made by private vehicles.^17^,^18^ In addition, greater compactness of residential developments would help preserve surrounding areas, which provide people with fundamental ecosystem services such as water and food.^19^ Neighborhood green spaces also have a number of environmental benefits: lowering ground temperature in the summer (mitigating heat islands), filtering airborne contaminants, and decreasing stormwater run-off.^20^ Good pedestrian infrastructure and green environments would also contribute to lower vehicle use by making active travel choices easier and attractive.

It is thus highly likely that environments designed to improve sustainability will also help make residents’ lifestyles more physically active. This is largely due to a common element in both goals (active lifestyles and sustainability); reduction of the dependency on private vehicles. Less automobile use leads to more physical activity for transport, less time for sitting while driving, less energy use, and less air pollution. Better air quality is another mechanism through which such environments enhance people’s health.^18^,^21^ Green spaces in neighborhoods, which contribute to environmental health, are also conducive to activity and contact with nature. Thus, it can be argued that environmental changes to improve sustainability may bring health benefits to residents in many different ways.

## Example: Shared Space

One example that may be employed to achieve the objectives discussed in this article is the idea of “shared space” (by pedestrians and motor vehicles). A traditional approach of traffic management is to divide streets into space for motor vehicles (road) and that for pedestrians (sidewalk). The principle of shared space is to integrate these spaces into one, where no space is clearly marked for vehicles.^22^ In such spaces, vehicles need to exercise extra caution to move across, and the priority is given to pedestrians. The street network of Bendigo city centre (Victoria, Australia) has been recently renovated using this principle.^23^ This substantial environmental change was planned partly to make the city centre more attractive and safe, which is expected to contribute to city’s economy. However, its immediate goal was to facilitate walking and deter vehicle use. Shared space applied to residential areas, which is called Home Zones, also aims to give pedestrians priority over vehicles.^24^ The idea of shared space is thus one example that could enhance both human health and sustainability. However, due to lack of formal evaluation, it is unknown to what extent this design principle can increase walking or decrease time spent sitting in vehicle.

## Conclusion

Research is needed to examine whether environments designed to enhance sustainability, such as shared space, compact city and smart growth, actually contribute to more physically-active and less sedentary lifestyles among residents. In spite that many environmental changes aiming for better sustainability are taking place, no research has investigated whether they are effective in changing people’s behavior patterns. At this stage, health benefits of sustainable environments are not yet confirmed. We need empirical research to corroborate to what extent, and through what mechanisms, sustainable environments contribute to people’s health.

However, conducting such research poses some significant challenges. Researchers have to know types of development and its location well before actual construction begins, so that they can arrange “baseline” data collection. To overcome this difficulty, it is important to establish a closer partnership between health researchers and those who are involved in planning, transport, development, and recreation in the public and private sectors. A single important step would be to incorporate physical activity assessments in environmental projects, where behavior change is expected to occur. Public health officials may play a key role in facilitating the introduction of such assessments by advocating that environmental changes could be a great opportunity for the promotion of population health. The findings of studies carried out in collaboration with the public or private sector would provide empirical support for developing policy and practice that aim to enhance human and environmental health simultaneously.

## References

[1] Sallis JE, Cervero RB, Ascher W, Henderson KA, Kraft MK, Kerr J (2006). An ecological approach to creating active living communities. Annual Review of Public Health.

[2] Bauman AE (2004). Updating the evidence that physical activity is good for health: An epidemiological review 2000–2003. Journal of Science and Medicine in Sport.

[3] Sallis JF, Linton L, Kraft MK (2005). The first active living research conference: Growth of a transdisciplinary field. American Journal of Preventive Medicine.

[4] (1996). US Department of Human Health Services Physical activity and health: A report of the Surgeon General.

[5] Australian Bureau of Statistics Physical activity in Australia: A snapshot, 2004–05, 2006.

[6] World Health Organization (2008). Global strategy on diet, physical activity and health. http://www.who.int/dietphysicalactivity/pa/en/index.html.

[7] Brownson RC, Boehmer TK, Luke DA (2005). Declining rates of physical activity in the United States: What are the contributors. Annual Review of Public Health.

[8] Dunstan DW, Salmon J, Owen N (2005). Associations of TV viewing and physical activity with the metabolic syndrome in Australian adults. Diabetologia.

[9] Frank LD, Andresen MA, Schmid TL (2004). Obesity relationships with community design, physical activity, and time spent in cars. American Journal of Preventive Medicine.

[10] Sugiyama T, Healy GN, Dunstan DW, Salmon J, Owen N (2008). Joint associations of multiple leisure-time sedentary behaviours and physical activity with obesity in Australian adults. International Journal of Behavioral Nutrition and Physical Activity.

[11] Marcus BH, Dubbert PM, Forsyth LH (2000). Physical activity behavior change: Issues in adoption and maintenance. Health Psychology.

[12] Cerin E, Leslie E, du Toit L, Owen N, Frank LD (2007). Destinations that matter: Associations with walking for transport. Health and Place.

[13] Aytur SA, Rodriguez DA, Evenson KR, Catellier DJ, Rosamond WD (2007). Promoting active community environments through land use and transportation planning. American Journal of Health Promotion.

[14] Santos R, Silva P, Santos P, Ribeiro JC, Mota J (2008). Physical activity and perceived environmental attributes in a sample of Portuguese adults: Results from the Azorean Physical Activity and Health Study. Preventive Medicine.

[15] Grahn P, Stigsdotter UA (2003). Landscape planning and stress. Urban Forestry and Urban Greening.

[16] Hartig T, Evans GW, Jamner LD, Davis DS, Garling T (2003). Tracking restoration in natural and urban field settings. Journal of Environmental Psychology.

[17] Jabareen YR (2006). Sustainable urban forms—Their typologies, models, and concepts. Journal of Planning Education and Research.

[18] Frank LD, Sallis JF, Conway TL, Chapman JE, Saelens BE, Bachman W (2006). Many pathways from land use to health: Associations between neighborhood walkability and active transportation, body mass index, and air quality. Journal of the American Planning Association.

[19] McDonald RI (2008). Global urbanization: Can. ecologists identify a sustainable way forward. Frontiers in Ecology and the Environment.

[20] Beer A, Delshammar T, Schildwacht P (2003). A changing understanding of the role of greenspace in high-density housing: A European perspective. Built Environment.

[21] Samet J, Krewski D (2007). Health effects associated with exposure to ambient air pollution. Journal of Toxicology and Environmental Health: Part A: Current Issues.

[22] Tolley R (2007). Shared space in Bendigo CBD: Principles, best practice and proposals: A report for presentation and assets, City of Greater Bendigo.

[23] Lucas C (2007). Walkers first on naked streets. Sydney Morning Herald.

[24] Barrell J, Whitehouse J (2004). Home Zones: An evolving approach to community streets. Proceedings of the Institution of Civil Engineers-Municipal Engineer.

